# Supplementary material for: The interactome of intact mitochondria by cross-linking mass spectrometry provides evidence for coexisting respiratory supercomplexes
Source: Mol Cell Proteomics. 2017 Dec 8;17(2):216–32. doi: 10.1074/mcp.RA117.000470 (PMC5795388; doi:10.1074/mcp.RA117.000470)
Supplement: Supplemental Data [file supp_RA117.000470_133922_0_supp_23978_fzffwf.zip › spectra_annotation/mito_spectra_annotation/328-1-6-1-10-1.pdf]

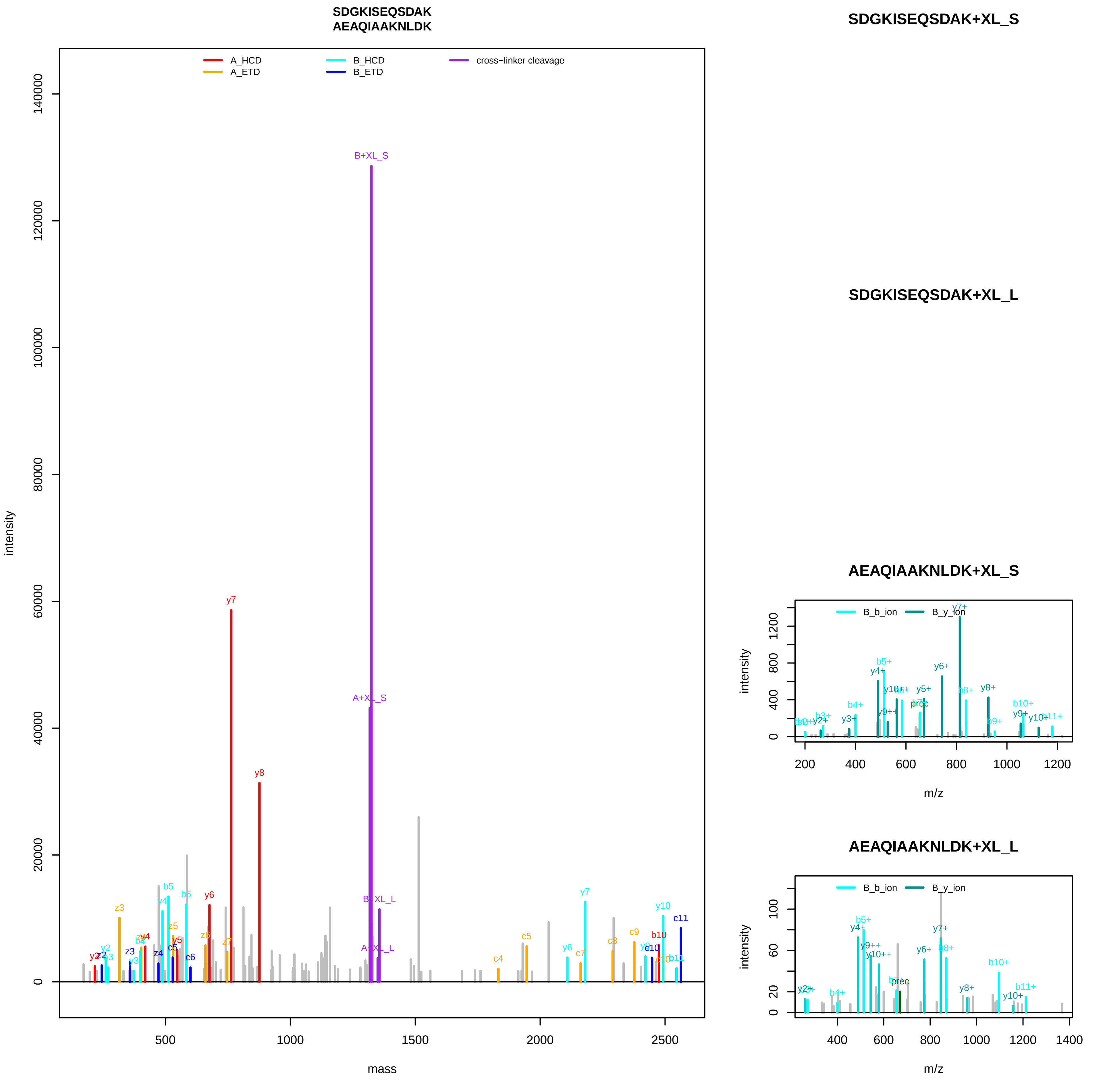

SDGKISEQSDAK
AEAQIAAKNLDK
A_HCD
A_ETD
B_HCD
B_ETD
cross−linker cleavage
B+XL_S
A+XL_S
B+XL_L
A+XL_L
y7
y8
y6
y5
y4
y2
z3
z5
z6
z7
c5
c6
c7
c8
c9
c10
b10
c11
y10
b11
b5
b6
y6
intensity
mass
0
20000
40000
60000
80000
100000
120000
140000
500
1000
1500
2000
2500
SDGKISEQSDAK+XL_S
SDGKISEQSDAK+XL_L
AEAQIAAKNLDK+XL_S
B_b_ion
B_y_ion
y7+
y6+
b5+
y4+
b8+
y8+
b10+
y9+
y10+
b11+
b4+
y3+
b3+
y2+
prec
intensity
m/z
0
400
800
1200
200
400
600
800
1000
1200
AEAQIAAKNLDK+XL_L
B_b_ion
B_y_ion
b5+
y4+
y9++
y10++
y7+
y6+
b8+
b10+
y8+
b11+
y10+
b4+
y2+
prec
intensity
m/z
0
20
60
100
400
600
800
1000
1200
1400
